# Presenilins and mitochondria—an intriguing link: mini-review

**DOI:** 10.3389/fnins.2023.1249815

**Published:** 2023-07-28

**Authors:** Mark Makarov, Liliia Kushnireva, Michele Papa, Eduard Korkotian

**Affiliations:** ^1^Department of Neurobiology, Weizmann Institute of Science, Rehovot, Israel; ^2^Department of Mental and Physical Health and Preventive Medicine, University of Campania "Luigi Vanvitelli", Caserta, Italy

**Keywords:** presenilins, Alzheimer’s disease, mitochondria associated membranes, spine apparatus, neurodegeneration

## Abstract

This review uncovers the intricate relationship between presenilins, calcium, and mitochondria in the context of Alzheimer’s disease (AD), with a particular focus on the involvement of presenilin mutations in mitochondrial dysfunction. So far, it is unclear whether the impairment of mitochondrial function arises primarily from damage inflicted by *β*-amyloid upon mitochondria or from the disruption of calcium homeostasis due to presenilins dysfunctions. The roles of presenilins in mitophagy, autophagy, mitochondrial dynamics, and many other functions, non-*γ*-secretase related, also require close attention in future research. Resolution of contradictions in understanding of presenilins cellular functions are needed for new effective therapeutic strategies for AD.

## Introduction

Presenilins (PS) are a family of highly conserved transmembrane proteins located in endoplasmic reticulum (ER), endosomes, and lysosomes, at ER-mitochondria contact sites, nuclear envelope, Golgi apparatus, but primarily in compartments near the cell surface (Area-Gomez et al., 2009; Escamilla-Ayala et al., 2020; Hernandez-Sapiens et al., 2022). The two homologs of PS, PS-1, and PS-2 are expressed ubiquitously in the brain and peripheral tissue, but have both overlapping and unique functions. PS is the catalytic core of the *γ*-secretase complex, which performs intramembrane proteolytic removal of many transmembrane proteins, including Notch-1, CD44, Syndecan, Delta, Jagged, and ErbB4, involved in various pathological processes (Escamilla-Ayala et al., 2020; Hernandez-Sapiens et al., 2022). However, presenilins are primarily known for their role in sequentially cleaving the amyloid precursor protein (APP) to generate *β*-amyloid (A*β*) peptides of various lengths. There are over 300 mutations in the presenilins genes, many of which result in excessive production of the most hydrophobic/aggregate form of A*β*42, leading to neurodegeneration and dementia in familial Alzheimer’s disease (FAD). Other mutations, as PS1 L286V or M146V and PS2 N141I, may increase the likelihood of apoptosis independently of amyloid via mitochondrial deficiency or calcium imbalance (Czech et al., 2000; Filadi and Pizzo, 2019). In addition to the amyloid hypothesis, various other theories have been proposed to explain the pathogenesis of Alzheimer’s disease (AD), including those involving tau protein accumulation, cholinergic dysfunction, viral infections, neuroinflammation, and the mitochondrial cascade hypothesis (Liu et al., 2019). Ca^2+^ signaling dysregulation emerges as a common underlying factor linking most of these theories (Filadi and Pizzo, 2019).

## The mitochondrial cascade hypothesis

Of particular interest, the mitochondrial cascade hypothesis has prompted investigations of the impact of presenilin mutations on *γ*-secretase activity, not only in relation to amyloidogenic processing, but also with regard to non-amyloid substrates and γ-secretase-independent functions of presenilins. These diverse functions encompass the modulation of calcium homeostasis, mitochondrial function, autophagy, and mitophagy. The hypothesis suggests a relative primacy of mitochondrial dysfunction over amyloid accumulation in the pathogenesis of AD and is currently subject to debate (Makarov and Korkotian, 2023). While the deleterious effects of amyloid accumulation are well-established, the question arises as to whether a single amyloid accumulation event is sufficient to elicit subsequent pathological consequences, and whether the amyloid hypothesis is excessively emphasized. Disruption of mitochondrial function due to impaired mitophagy may represent a more pivotal mechanism. It has been documented that compromised mitochondrial function promotes increased production of Aβ in AD models (Leuner et al., 2012), while induction of mitophagy has shown potential for alleviating disease manifestations (Lautrup et al., 2019; Lee et al., 2021). Presenilins participate in mitophagy through various pathways, including interaction with PINK1/Parkin proteins for mitophagy regulation (Quinn et al., 2020; Saito et al., 2022), and control of gene expression associated with PARK2-dependent autophagy through transcription factor interactions (Checler et al., 2017). Presenilins also exert influence on the regulation of autophagy. For instance, mutated PS2 has been found to impede autophagy at the stage of autophagosome-lysosome fusion, attributed to disruptions in Ca^2+^ homeostasis (Fedeli et al., 2019). The elucidation of the precise role of presenilins in mitophagy and autophagy represents a dynamic area of investigation, with potential implications for advancing comprehension of AD pathogenesis as well as the broader cellular functions of presenilins.

## Effect of presenilins on mitochondrial dynamic

The functional roles of presenilins extend to the regulation of mitochondrial dynamics, encompassing processes, such as movement, fusion, fission, and distribution within neurons (Han et al., 2021). Precise and coordinated movement of mitochondria in both anterograde and retrograde directions is crucial for fulfilling the metabolic demands of cell regions, particularly those located distally from the soma, such as axon terminals and distal dendrites. Significantly, specific PS1 mutations, including M146V, I143T, and D9, have been found to impair kinesin-based anterograde axonal transport by inducing excessive activation of glycogen synthase kinase 3*β* (GSK3*β*), which plays a key role in the transmission of regulatory and proliferative signals in cells. Increased phosphorylation of kinesin light chain (KLC) by GSK3*β* promotes the dissociation of kinesin-I from membrane-bound organelles (MBOs), leading to diminished motility of mitochondria and synaptic vesicles. In fact, cultured hippocampal neurons expressing the PS1 M146V mutation exhibited reduced density of both mitochondria and synaptic vesicles (Wang et al., 2020). It has been established that the anterograde transport of mitochondria is the most vulnerable to A*β* oligomers (Correia et al., 2016). However, the precise consequences of presenilin mutations on the intricate anterograde transport process mediated by kinesin motors in dendrites have yet to be fully elucidated. Additionally, PS1 mutations also disrupt retrograde transport—a critical mechanism for sustaining synaptic plasticity and facilitating the removal of damaged mitochondria. The impairment of dynein-mediated transport, responsible for the transportation of cargo proteins to the soma for degradation, leads to the formation of obstructions, thereby compromising neuronal signaling and the transport of molecules and organelles (Kimura et al., 2012).

The process of mitochondrial fusion plays a crucial role in maintaining plasticity mechanisms, as well as in response to stress conditions. Mitochondrial “fusion” is a physical connection of the outer and inner mitochondrial membranes of two initially separate mitochondria promotes enhanced oxidative phosphorylation processes and dilution of damaged mitochondrial DNA (Gao and Hu, 2021). Conversely, the “fission” mechanism is in which mitochondria divide into two separate organelles serves to generate new mitochondria and aid in the removal of impaired ones. In AD, a notable increase in fragmented mitochondria is observed, which is associated with mutations in presenilins. Post-mortem analyses of AD patient tissues frequently exhibit elevated levels of dynamin-related protein 1 (Drp1), a key protein involved in mitochondrial fission, along with decreased expression of fusion proteins. While Drp1-mediated fission is essential for maintaining mitochondrial function and facilitating their sequestration within autophagosomes, excessive fission may disrupt their interaction with the endoplasmic reticulum (ER) at mitochondria-associated membranes (MAMs; Shields et al., 2021). Drp1-caused overfission leads to abnormally small mitochondria with impaired bioenergetics: the ability to buffer calcium and produce ATP, which contributes to synapse damage and further loss of neurons (Wang et al., 2020).

## The role of presenilins in calcium homeostasis

Presenilins also exhibit localization in MAM contact sites, where they play a direct role in mediating communication and calcium transfer between ER and mitochondria. Mechanisms underlying the impact of presenilin mutations on MAM contacts, ER-mitochondrial crosstalk, and mitochondrial reactive oxygen species (ROS) production remain poorly understood (Hedskog et al., 2013). Studies using primary cortical neurons from PS1 knockout mice have demonstrated elevated calcium release from the ER mediated by inositol 1,4,5-trisphosphate (IP3) receptors. PS1 M146L and PS2 N141I mutations prolong the opening time of IP3 channels, leading to increased Ca^2+^ leakage permeability (Cheung et al., 2008). Number and function of ryanodine receptors (RyR) are also increased in mouse models containing mutations PS1 M146V and PS2 N141I (Supnet and Bezprozvanny, 2011). At the same time, there is evidence suggesting that transmembrane domains 7 and 9 of PS1 have been implicated in pore-forming of an ionic conduction as Ca^2+^ − leak channel (Zhang et al., 2013). However, the PS1-M146V, PS2-N141I, and some others mutations disrupt or abolish this leak, resulting in ER Ca^2+^ overload (Nelson et al., 2007). The optimal width of the MAM is crucial for the proper transport of Ca2+ between the ER and mitochondria (Zhang et al., 2021). A reduction in the distance between the ER and mitochondria within MAM regions has been observed in the context of Alzheimer’s disease, potentially serving as a compensatory mechanism resulting from diminished Ca2+ leakage (Perez-Leanos et al., 2021). Furthermore, presenilins are also capable of physically interacting with the sarco/endoplasmic reticulum Ca^2+^ ATPase (SERCA), which actively transports Ca^2+^ from the cytosol to the ER, thereby helping to maintain low resting levels of cytosolic Ca^2+^. PS2-mediated calcium sequestration by SERCA pumps is vital for refilling ER calcium stores (Green et al., 2008; Fedeli et al., 2019).

## Presenilin mutations dysregulate mitochondria with calcium

Paradoxically, while evidence indicates that loss of leakage function of presenilins may lead to Ca^2+^ ER overload, both overexpression of normal presenilins and some FAD-related mutations in them have been associated with increased Ca^2+^ release from the ER, depletion of Ca^2+^ stores, and an increase in cytosolic Ca^2+^ (Nelson et al., 2007; Galla et al., 2020). Other FAD mutations, on the contrary, reduce IP3-mediated increase in cytosolic Ca^2+^ (Galla et al., 2020). Dysregulation of calcium signaling observed in both scenarios has deleterious consequences for mitochondrial energy functions (Korkotian et al., 2019). Decreased Ca^2+^ ER-leakage caused by presenilin mutants leads to ER overload and hence exaggerated Ca^2+^ release upon cell stimulation (Zampese et al., 2011). By increasing Ca^2+^ release through the ER membrane, PS1 mutations can enhance Ca^2+^ signaling at synaptic terminals, leading to dysfunction and degeneration in aging and amyloid accumulation in AD (Mattson, 2010).

Presenilins play a vital role in mediating interactions between the mitochondria-associated endoplasmic reticulum (ER) membranes, with a particular emphasis in PS2. PS2 functions as a positive regulator of the calcium connection between ER and mitochondria (Filadi et al., 2016; Korkotian et al., 2019). The compromised signaling of local cytosolic Ca^2+^ resulting from the aforementioned overload of calcium stores within the ER and spine apparatus, can diminish the capacity to attract, immobilize, and retain mitochondria beneath synapses that exhibit high ATP demand. This, in turn, leads to a diminished ability to form stable synapses. Conversely, excessive levels of calcium transferred from the ER to mitochondria-associated ER membranes can give rise to mitochondrial Ca^2+^ overload, leading to the decoupling of the oxidative phosphorylation chain and subsequent reduction in ATP production (Kushnireva and Korkotian, 2022). Strikingly, some studies report that mitochondrial Ca^2+^ uptake in FAD-linked PS2 mutants is reduced as a result of decreased ER Ca^2+^, causing a blunted increase in cytosolic Ca^2+^ after stimulation (Rossini et al., 2021). These effects also depend on the ability of PS2 to partially block SERCA activity and induce hyperactivation of IP3 receptors (Rossini et al., 2021).

**Figure 1:**
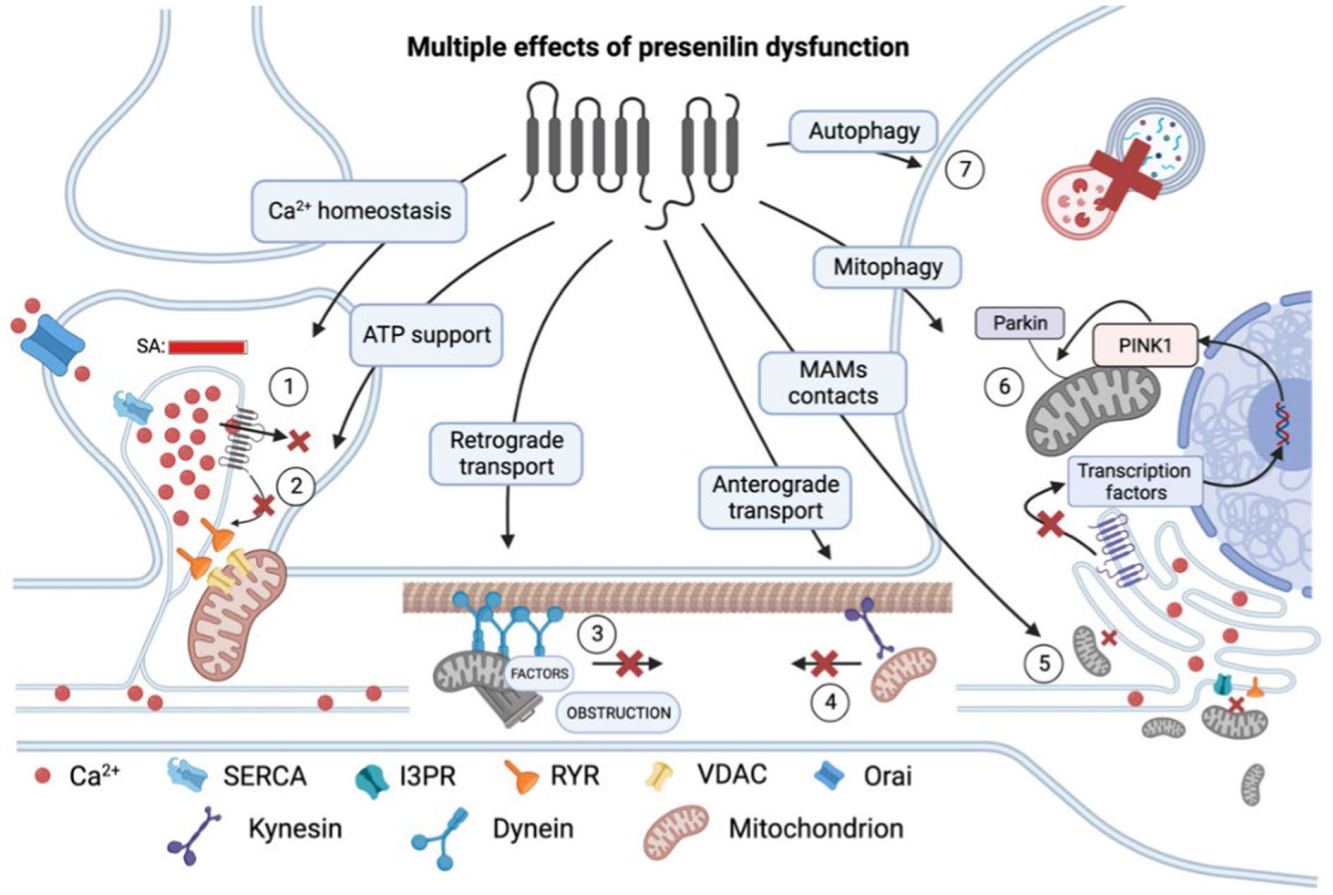
Multiple effects of presenilin (PS) dysfunction. Presenilin mutations result in an overload of calcium (Ca^2+^) within the spine apparatus (SA) due to decreased leakage via (1) pore of PS itself and (2) reduced influence of PS on RYR channels. This excessive calcium accumulation leads to impaired calcium conduction into mitochondria and decreased ATP production and therefore energy support of postsynaptic processes. (3) Presenilin mutations disrupt retrograde transport and cause dendrite obstruction. (4) Presenilin mutations lead to a reduction in connections between kinesin and membrane-bound organelles, such as mitochondria. (5) Mutations in presenilin result in extensive mitochondrial fission and impaired mitochondria-ER contact at mitochondria-associated membranes (MAMs). (6) Presenilin influences mitophagy by participating in the activation of transcription factors of PINK1 genes, which are essential for recruiting ubiquitin ligase PARKIN to the mitochondrial membrane and establishing a connection with the phagophore membrane. (7) Dysregulation of calcium homeostasis hampers autophagy by impairing lysosomal fusion. RYR, ryanodine receptor; SERCA, sarco/endoplasmic reticulum Ca^2+^-ATPase; and VDAC, voltage-dependent anion channels. In the figure, a red cross denotes the inhibition of a process.

An increased number of MAM sites has been observed in cellular and animal models of AD as a compensatory mechanism against decreased ER-Ca^2+^ for improving Ca^2+^ transfer from ER to mitochondria (Green et al., 2008), as well as result of accumulation of C99 and ceramides (Pera et al., 2017). In addition, the presence of FAD-linked PS2 mutations leads to increased juxtaposition of ER-mitochondria (Zampese et al., 2011). However, the coexisting exaggerated release of Ca^2+^ via RyR- and IP3 receptors flowing into mitochondria stimulates oxidative phosphorylation, which leads to increased production of ROS, mitochondrial degradation, and neurodegeneration (Toglia et al., 2016; Ryan et al., 2020). This same excess release that increases cytosolic calcium levels also interferes with the induction of long-term potentiation and mediates some of the cytotoxic effects of *β*-amyloid (Yu et al., 2021). Thus, the impairment of calcium homeostasis induced by presenilins affects the development of neurodegeneration in many controversial and unclear ways. The most vulnerable in this case are mitochondrial functions, including those associated with their maintenance of synaptic plasticity. Calcium dysregulation and mitochondrial injury can be claimed to be causative factors of neurodegeneration in AD, being common mechanisms implicated in the onset of both genetic and sporadic forms of this age-related pathology (Figure 1).

## Presenilin in astrocytes

The association between presenilins and Alzheimer’s disease extends beyond their impact on neurons, as emerging evidence suggests their involvement in astrocytes and the perisynaptic region of tripartite synapses. Postulated that astrocytes, when damaged, release inflammatory factors that contribute to the progression of neurodegeneration, with gliosis being an early symptom (Carter et al., 2012; Virtuoso et al., 2023). Astrocytes may also play a role in synaptic loss, as studies have shown an association between PS1&PS2 knockout and astrogliosis resulting in neuroinflammation (Peng et al., 2022). The effect of presenilin in astrocytic mitochondria remains inconclusive, as Contino et al. (2021) found no influence on the mitochondria of cultured neurons and astrocytes. However, presenilin deficiency has been shown to induce hyperfunction of the mitochondria-associated endoplasmic reticulum membranes (MAMs). In this regard, MAM-mediated synthesis of phospholipids and cholesterol significantly increased in astrocytes treated with apolipoprotein E, which is implicated in Alzheimer’s disease (Tambini et al., 2016).

## Discussion

The primary cause of mitochondrial dysfunction in the pathogenesis of FAD remains elusive. Specifically, it is unclear whether the impairment of mitochondrial function arises primarily from damage inflicted by β-amyloid upon mitochondria or from the disruption of calcium homeostasis due to the dysfunctional presenilins. The independent role of presenilins in calcium homeostasis and their effect on the quality of mitochondria also requires deep study. In the article, we have mentioned the accumulated contradictions regarding the mechanism of the influence of mutant presenilins on ATP synthesis through disruption of the ER, SA, and MAM in particular. The reason for these contradictions can be both the use of calcium indicators with different sensitivities in experiments, and the variability of the multiple established effects at rest and in cellular activation. Connections of certain presenilin mutations with the impaired local regulation of intracellular calcium in SA (which is functionally different from the ER), will help to resolve the existing contradictions and identify the root causes of mitochondrial dysfunction in neurodegeneration. Accurate determination of the role played by presenilin in the mitochondrial cascade would provide valuable guidance to researchers in their quest for therapeutic targets and the development of treatment and prevention strategies.

While the role of mutant presenilins is unquestionable, it remains uncertain whether their presence alone is sufficient and which factors contribute to the implementation of FAD. Disturbances in calcium homeostasis and associated mitochondrial dysfunction are anticipated to have significant implications in aging and degeneration, making it an area of paramount importance for research. Additionally, further elucidation is necessary regarding the multiple functions of presenilins in various brain structures and cell types that may contribute to particular mitochondrial dynamics and bioenergetics. Given the apparent failure of drug therapies targeting at amyloid peptide clearance, it is imperative to focus on alternative hypotheses regarding the etiology of AD to identify effective therapeutic interventions.

## Author contributions

EK: conceptualization and writing-review and editing. MM and LK: literature research and analysis and writing. MM: visualization. EK and MP: supervision. All authors contributed to the article and approved the submitted version.

## Funding

This work was supported by #NEXTGENERATIONEU (NGEU) and funded by the Ministry of University and Research (MUR), National Recovery and Resilience Plan (NRRP), project MNESYS (PE0000006)—A Multiscale integrated approach to the study of the nervous system in health and disease (DN. 1553 11.10.2022).

## Conflict of interest

The authors declare that the research was conducted in the absence of any commercial or financial relationships that could be construed as a potential conflict of interest.

## Publisher’s note

All claims expressed in this article are solely those of the authors and do not necessarily represent those of their affiliated organizations, or those of the publisher, the editors and the reviewers. Any product that may be evaluated in this article, or claim that may be made by its manufacturer, is not guaranteed or endorsed by the publisher.

## References

[ref1] Area-Gomez E. de Groof A. J. Boldogh I. Bird T. D. Gibson G. E. Koehler C. M.. (2009). Presenilins are enriched in endoplasmic reticulum membranes associated with mitochondria. Am. J. Pathol. 175, 1810–1816. doi: 10.2353/ajpath.2009.090219, PMID: 19834068 PMC2774047

[ref2] Carter B. S. Meng F. Thompson R. C. (2012). Glucocorticoid treatment of astrocytes results in temporally dynamic transcriptome regulation and astrocyte-enriched mRNA changes in vitro. Physiol. Genomics 44, 1188–1200. doi: 10.1152/physiolgenomics.00097.2012, PMID: 23110767 PMC3544487

[ref3] Checler F. Goiran T. Alves da Costa C. (2017). Presenilins at the crossroad of a functional interplay between PARK2/PARKIN and PINK1 to control mitophagy: implication for neurodegenerative diseases. Autophagy 13, 2004–2005. doi: 10.1080/15548627.2017.1363950, PMID: 28914586 PMC5788489

[ref4] Cheung K. H. Shineman D. Müller M. Cardenas C. Mei L. Yang J.. (2008). Mechanism of Ca2+ disruption in Alzheimer's disease by presenilin regulation of InsP3 receptor channel gating. Neuron 58, 871–883. doi: 10.1016/j.neuron.2008.04.015, PMID: 18579078 PMC2495086

[ref5] Contino S. Suelves N. Vrancx C. Vadukul D. M. Payen V. L. Stanga S.. (2021). Presenilin-deficient neurons and astrocytes display normal mitochondrial phenotypes. Front. Neurosci. 14:586108. doi: 10.3389/fnins.2020.586108, PMID: 33551720 PMC7862347

[ref6] Correia S. C. Perry G. Moreira P. I. (2016). Mitochondrial traffic jams in Alzheimer's disease—pinpointing the roadblocks. Biochim. Biophys. Acta 1862, 1909–1917. doi: 10.1016/j.bbadis.2016.07.010, PMID: 27460705

[ref7] Czech C. Tremp G. Pradier L. (2000). Presenilins and Alzheimer's disease: biological functions and pathogenic mechanisms. Prog. Neurobiol. 60, 363–384. doi: 10.1016/s0301-0082(99)00033-7, PMID: 10670705

[ref8] Escamilla-Ayala A. A. Sannerud R. Mondin M. Poersch K. Vermeire W. Paparelli L.. (2020). Super-resolution microscopy reveals majorly mono- and dimeric presenilin1/γ-secretase at the cell surface. elife 9:e56679. doi: 10.7554/eLife.56679, PMID: 32631487 PMC7340497

[ref9] Fedeli C. Filadi R. Rossi A. Mammucari C. Pizzo P. (2019). PSEN2 (presenilin 2) mutants linked to familial Alzheimer disease impair autophagy by altering Ca2+ homeostasis. Autophagy 15, 2044–2062. doi: 10.1080/15548627.2019.1596489, PMID: 30892128 PMC6844518

[ref10] Filadi R. Greotti E. Turacchio G. Luini A. Pozzan T. Pizzo P. (2016). Presenilin 2 modulates endoplasmic reticulum-mitochondria coupling by tuning the antagonistic effect of Mitofusin 2. Cell Rep. 15, 2226–2238. doi: 10.1016/j.celrep.2016.05.013, PMID: 27239030

[ref11] Filadi R. Pizzo P. (2019). Defective autophagy and Alzheimer's disease: is calcium the key? Neural Regen. Res. 14, 2081–2082. doi: 10.4103/1673-5374.262584, PMID: 31397341 PMC6788238

[ref12] Galla L. Redolfi N. Pozzan T. Pizzo P. Greotti E. (2020). Intracellular calcium dysregulation by the Alzheimer's disease-linked protein Presenilin 2. Int. J. Mol. Sci. 21:770. doi: 10.3390/ijms21030770, PMID: 31991578 PMC7037278

[ref13] Gao S. Hu J. (2021). Mitochondrial fusion: the machineries in and out. Trends Cell Biol. 31, 62–74. doi: 10.1016/j.tcb.2020.09.008 33092941

[ref14] Green K. N. Demuro A. Akbari Y. Hitt B. D. Smith I. F. Parker I.. (2008). SERCA pump activity is physiologically regulated by presenilin and regulates amyloid β production. J. Cell Biol. 181, 1107–1116. doi: 10.1083/jcb.200706171, PMID: 18591429 PMC2442205

[ref15] Han J. Park H. Maharana C. Gwon A. R. Park J. Baek S. H.. (2021). Alzheimer's disease-causing presenilin-1 mutations have deleterious effects on mitochondrial function. Theranostics 11, 8855–8873. doi: 10.7150/thno.59776, PMID: 34522215 PMC8419044

[ref16] Hedskog L. Pinho C. M. Filadi R. Rönnbäck A. Hertwig L. Wiehager B.. (2013). Modulation of the endoplasmic reticulum-mitochondria interface in Alzheimer's disease and related models. Proc. Natl. Acad. Sci. U. S. A. 110, 7916–7921. doi: 10.1073/pnas.1300677110, PMID: 23620518 PMC3651455

[ref17] Hernandez-Sapiens M. A. Reza-Zaldívar E. E. Márquez-Aguirre A. L. Gómez-Pinedo U. Matias-Guiu J. Cevallos R. R.. (2022). Presenilin mutations and their impact on neuronal differentiation in Alzheimer's disease. Neural Regen. Res. 17, 31–37. doi: 10.4103/1673-5374.313016, PMID: 34100423 PMC8451546

[ref18] Kimura N. Okabayashi S. Ono F. (2012). Dynein dysfunction disrupts intracellular vesicle trafficking bidirectionally and perturbs synaptic vesicle docking via endocytic disturbances a potential mechanism underlying age-dependent impairment of cognitive function. Am. J. Pathol. 180, 550–561. doi: 10.1016/j.ajpath.2011.10.037, PMID: 22182700

[ref19] Korkotian E. Meshcheriakova A. Segal M. (2019). Presenilin 1 regulates [Ca2+]i and mitochondria/ER interaction in cultured rat hippocampal neurons. Oxidative Med. Cell. Longev. 2019, 7284914–7284967. doi: 10.1155/2019/7284967, PMID: 31467635 PMC6701405

[ref20] Kushnireva L. Korkotian E. (2022). Mitochondria-Endoplasmic Reticulum Interaction in Central Neurons In Updates on Endoplasmic Reticulum. London, UK: IntechOpen.

[ref21] Lautrup S. Lou G. Aman Y. Nilsen H. Tao J. Fang E. F. (2019). Microglial mitophagy mitigates neuroinflammation in Alzheimer's disease. Neurochem. Int. 129:104469. doi: 10.1016/j.neuint.2019.104469, PMID: 31100304

[ref22] Lee H. J. Jung Y. H. Choi G. E. Kim J. S. Chae C. W. Lim J. R.. (2021). Urolithin a suppresses high glucose-induced neuronal amyloidogenesis by modulating TGM2-dependent ER-mitochondria contacts and calcium homeostasis. Cell Death Differ. 28, 184–202. doi: 10.1038/s41418-020-0593-1, PMID: 32704090 PMC7852667

[ref23] Leuner K. Schütt T. Kurz C. Eckert S. H. Schiller C. Occhipinti A.. (2012). Mitochondrion-derived reactive oxygen species lead to enhanced amyloid beta formation. Antioxid. Redox Signal. 16, 1421–1433. doi: 10.1089/ars.2011.4173, PMID: 22229260 PMC3329950

[ref24] Liu P. P. Xie Y. Meng X. Y. Kang J. S. (2019). History and progress of hypotheses and clinical trials for Alzheimer's disease. Signal Transduct. Target. Ther. 4:29. doi: 10.1038/s41392-019-0063-8, PMID: 31637009 PMC6799833

[ref25] Makarov M. Korkotian E. (2023). Differential role of active compounds in Mitophagy and related neurodegenerative diseases. Toxins 15:202. doi: 10.3390/toxins15030202, PMID: 36977093 PMC10058020

[ref26] Mattson M. P. (2010). ER calcium and Alzheimer's disease: in a state of flux. Sci. Signal. 3:pe10. doi: 10.1126/scisignal.3114pe10, PMID: 20332425 PMC3091478

[ref27] Nelson O. Tu H. Lei T. Bentahir M. De Strooper B. Bezprozvanny I. (2007). Familial Alzheimer disease–linked mutations specifically disrupt ca 2+ leak function of presenilin 1. J. Clin. Invest. 117, 1230–1239. doi: 10.1172/JCI30447 17431506 PMC1847535

[ref28] Peng W. Xie Y. Liao C. Bai Y. Wang H. Li C. (2022). Spatiotemporal patterns of gliosis and neuroinflammation in presenilin 1/2 conditional double knockout mice. Front. Aging Neurosci. 14:966153. doi: 10.3389/fnagi.2022.966153, PMID: 36185485 PMC9521545

[ref29] Pera M. Larrea D. Guardia-Laguarta C. Montesinos J. Velasco K. R. Agrawal R. R.. (2017). Increased localization of APP-C99 in mitochondria-associated ER membranes causes mitochondrial dysfunction in Alzheimer disease. EMBO J. 36, 3356–3371. doi: 10.15252/embj.201796797, PMID: 29018038 PMC5731665

[ref30] Perez-Leanos C. A. Romero-Campos H. E. Dupont G. Gonzalez-Velez V. (2021). Reduction of ER-mitochondria distance: a key feature in Alzheimer's and Parkinson's disease, and during Cancer treatment. Annu. Int. Conf. IEEE Eng. Med. Biol. Soc. 2021, 4412–4415. doi: 10.1109/EMBC46164.2021.9631090, PMID: 34892198

[ref31] Quinn P. M. J. Moreira P. I. Ambrósio A. F. Alves C. H. (2020). PINK1/PARKIN signalling in neurodegeneration and neuroinflammation. Acta Neuropathol. Commun. 8:189. doi: 10.1186/s40478-020-01062-w, PMID: 33168089 PMC7654589

[ref32] Rossini M. García-Casas P. Filadi R. Pizzo P. (2021). Loosening ER-mitochondria coupling by the expression of the Presenilin 2 loop domain. Cells 10:1968. doi: 10.3390/cells10081968, PMID: 34440738 PMC8394530

[ref33] Ryan K. C. Ashkavand Z. Norman K. R. (2020). The role of mitochondrial calcium homeostasis in Alzheimer's and related diseases. Int. J. Mol. Sci. 21:9153. doi: 10.3390/ijms21239153, PMID: 33271784 PMC7730848

[ref34] Saito T. Kodani E. Gottlieb R. A. (2022). Presenilin-2 regulates Mitophagy via promoting intracellular translocation of Parkin to mitochondria. Circulation 146:A12308. doi: 10.1161/circ.146.suppl_1.12308

[ref35] Shields L. Y. Li H. Nguyen K. Kim H. Doric Z. Garcia J. H.. (2021). Mitochondrial fission is a critical modulator of mutant APP-induced neural toxicity. J. Biol. Chem. 296:100469. doi: 10.1016/j.jbc.2021.100469, PMID: 33639169 PMC8042169

[ref36] Supnet C. Bezprozvanny I. (2011). Presenilins function in ER calcium leak and Alzheimer's disease pathogenesis. Cell Calcium 50, 303–309. doi: 10.1016/j.ceca.2011.05.013, PMID: 21663966 PMC3172403

[ref37] Tambini M. D. Pera M. Kanter E. Yang H. Guardia-Laguarta C. Holtzman D.. (2016). ApoE4 upregulates the activity of mitochondria-associated ER membranes. EMBO Rep. 17, 27–36. doi: 10.15252/embr.201540614, PMID: 26564908 PMC4718413

[ref38] Toglia P. Cheung K. H. Mak D. O. Ullah G. (2016). Impaired mitochondrial function due to familial Alzheimer's disease-causing presenilins mutants via ca(2+) disruptions. Cell Calcium 59, 240–250. doi: 10.1016/j.ceca.2016.02.013, PMID: 26971122 PMC5088788

[ref39] Virtuoso A. De Luca C. Korai S. A. Papa M. Cirillo G. (2023). Neuroinflammation and glial activation in the central nervous system: a metabolic perspective. Neural Regen. Res. 18, 1025–1026. doi: 10.4103/1673-5374.355754, PMID: 36254985 PMC9827792

[ref40] Wang W. Zhao F. Ma X. Perry G. Zhu X. (2020). Mitochondria dysfunction in the pathogenesis of Alzheimer’s disease: recent advances. Mol. Neurodegener. 15:30. doi: 10.1186/s13024-020-00376-6, PMID: 32471464 PMC7257174

[ref41] Yu W. Jin H. Huang Y. (2021). Mitochondria-associated membranes (MAMs): a potential therapeutic target for treating Alzheimer's disease. Clin. Sci. (Lond.) 135, 109–126. doi: 10.1042/CS20200844, PMID: 33404051 PMC7796309

[ref42] Zampese E. Fasolato C. Kipanyula M. J. Bortolozzi M. Pozzan T. Pizzo P. (2011). Presenilin 2 modulates endoplasmic reticulum (ER)-mitochondria interactions and Ca2+ cross-talk. Proc. Natl. Acad. Sci. U. S. A. 108, 2777–2782. doi: 10.1073/pnas.1100735108, PMID: 21285369 PMC3041131

[ref43] Zampese E. Fasolato C. Pozzan T. Pizzo P. (2011). Presenilin-2 modulation of ER-mitochondria interactions: FAD mutations, mechanisms and pathological consequences. Commun. Integr. Biol. 4, 357–360. doi: 10.4161/cib.4.3.15160, PMID: 21980580 PMC3187908

[ref44] Zhang S. Zhang M. Cai F. Song W. (2013). Biological function of Presenilin and its role in AD pathogenesis. Translat. Neurodegen. 2, 1–13. doi: 10.1186/2047-9158-2-15 PMC3718700 23866842

[ref45] Zhang S. S. Zhou S. Crowley-McHattan Z. J. Wang R. Y. Li J. P. (2021). A review of the role of Endo/sarcoplasmic reticulum-mitochondria Ca2+ transport in diseases and skeletal muscle function. Int. J. Environ. Res. Public Health 18:3874. doi: 10.3390/ijerph18083874, PMID: 33917091 PMC8067840

